# Universal HIV Screening at Postnatal Points of Care: Which Public Health Approach for Early Infant Diagnosis in Côte d'Ivoire?

**DOI:** 10.1371/journal.pone.0067996

**Published:** 2013-08-21

**Authors:** Camille Ndondoki, Hermann Brou, Marguerite Timite-Konan, Maxime Oga, Clarisse Amani-Bosse, Hervé Menan, Didier Ekouévi, Valériane Leroy

**Affiliations:** 1 Inserm, U897, Centre de Recherche Epidémiologie et Biostatistique, Institut de Santé Publique, Epidémiologie et Développement (ISPED), Université Bordeaux Victor Segalen, Bordeaux, France; 2 Programme PACCI, Projet Pedi-Test ANRS 12165, Abidjan, Côte d'Ivoire; 3 Service de Pédiatrie, CHU de Yopougon, Abidjan, Côte d'Ivoire; 4 Laboratoire de Virologie du CeDRes, Abidjan, Côte d'Ivoire; University of Cape Town, South Africa

## Abstract

**Background:**

Universal HIV pediatric screening offered at postnatal points of care (PPOC) is an entry point for early infant diagnosis (EID). We assessed the parents' acceptability of this approach in Abidjan, Côte d'Ivoire.

**Methods:**

In this cross-sectional study, trained counselors offered systematic HIV screening to all children aged 6–26 weeks attending PPOC in three community health centers with existing access to HAART during 2008, as well as their parents/caregivers. HIV-testing acceptability was measured for parents and children; rapid HIV tests were used for parents. Both parents' consent was required according to the Ivorian Ethical Committee to perform a HIV test on HIV-exposed children. Free HIV care was offered to those who were diagnosed HIV-infected.

**Findings:**

We provided 3,013 HIV tests for infants and their 2,986 mothers. While 1,731 mothers (58%) accepted the principle of EID, only 447 infants had formal parental consent 15%; 95% confidence interval (CI): [14%–16%]. Overall, 1,817 mothers (61%) accepted to test for HIV, of whom 81 were HIV-infected (4.5%; 95% CI: [3.5%–5.4%]). Among the 81 HIV-exposed children, 42 (52%) had provided parental consent and were tested: five were HIV-infected (11.9%; 95% CI: [2.1%–21.7%]). Only 46 fathers (2%) came to diagnose their child. Parental acceptance of EID was strongly correlated with prenatal self-reported HIV status: HIV-infected mothers were six times more likely to provide EID parental acceptance than mothers reporting unknown or negative prenatal HIV status (aOR: 5.9; 95% CI: [3.3–10.6], p = 0.0001).

**Conclusions:**

Although the principle of EID was moderately accepted by mothers, fathers' acceptance rate remained very low. Routine HIV screening of all infants was inefficient for EID at a community level in Abidjan in 2008. Our results suggest the need of focusing on increasing the PMTCT coverage, involving fathers and tracing children issued from PMTCT programs in low HIV prevalence countries.

## Introduction

The proportion of pregnant women who tested for HIV in sub-Sarahan Africa was 42% in 2010 [1]. Close to 50% of HIV infected pregnant women and 42% of their infants received antiretroviral (ARVs) for prevention of mother-to-child HIV transmission (PMTCT) [1]. Despite the efficacy of interventions for PMTCT, the HIV pediatric epidemic is still growing in the world, and 90% of HIV infected children live in Africa [2]–[4]. At the end of 2009, an estimated 370000 children [220000–520000] worldwide contracted HIV during the perinatal and breastfeeding period [5], [6]. Early and high mortality is observed among HIV infected children without antiretroviral therapy; up to 50% of them will die before their second birthday [4]. Despite this, children are less likely to receive antiretroviral therapy than the adult population: in 2009, among the 1.27 million children estimated in need of treatment in low income countries, 28% had access to antiretroviral treatment (versus 37% in adults) [6]; these children represent only 7% of people receiving antiretroviral therapy worldwide [6].

In 2008, the WHO recommended universal early antiretroviral treatment for HIV-infected children aged less than 12 months to improve their survival, irrespective of clinical status or symptomatology [7], [8]. In 2010, these guidelines were revised to include therapy for all children less than 24 months of age [9]. To improve early access to pediatric antiretroviral therapy in resource-limited settings, it is crucial to assess public health strategies for early HIV infant diagnosis (EID) at the community level [10].

While adult HIV counseling and testing practices have been scaled up in low income countries, the routine offering of HIV testing to pediatric patients in such settings often occurs too late [2], [11], [12]. Only 6% of infants born to HIV infected mothers had access to EID in 2009 [6]. EID including both children exposed and non-exposed to PMTCT interventions is the main operational challenge in Africa [13]. EID requires active investment in key areas such as training and support for providers, improvement of laboratory tools and referral networks. The use of dried blood spot (DBS) specimens collected on filter paper can improve testing networks for early infant diagnosis as in South Africa, but still needs to be generalized in many African countries [14], [15].

However, it is also important to understand how to efficiently reach all HIV exposed children. The feasibility of routine early HIV testing for infants as a public health strategy is highly conditioned by its social and familial acceptability. Few studies have explored public health strategies to identify perinatally HIV-exposed children among pediatric populations in high HIV prevalence settings in Eastern and Southern Africa [16]–[18].

In Côte d'Ivoire, the HIV prevalence among pregnant women was 3.4% in 2009 [19]. Close to 85% of pregnant women were engaged in prenatal care and 57% of them gave birth in hospital; 47% were HIV tested during gestation [20], [21], [22], The availability of antiretrovirals for PMTCT was estimated to 54% for HIV infected pregnant women and 33% of children born to HIV infected mothers received antiretroviral prophylaxis at birth. Close to 6% of children received antiretroviral therapy in 2009 [23]. HIV testing is part of the standard-of-care, in Côte d'Ivoire when children are known to be HIV-exposed or if they have symptoms suggesting HIV-infection. DBS collected on filter paper was used as a national strategy for children testing since 2008. Within this context, the PEDI-TEST Project ANRS 12165 aims to evaluate the parental acceptability of early HIV diagnosis of infants between 6–26 weeks, at postnatal points of care (PPOC) in Abidjan, Côte d'Ivoire.

## Methods

### Ethics statement

The study was approved by the Ivoirian Ethical Committee (March 2008). The National Ethics Committee, in reference to the law of the civil code n°70–453 of 3 August 1970 on minorities in Cote d'Ivoire, specified that “HIV-testing and care for children who are enrolled in any clinical research study must be conditioned by mutual and written informed consent of both their father and mother.”

### Study design and patients

The PEDI-TEST ANRS 12165 Study was a cross-sectional evaluation of both health care workers and family- acceptability of routine HIV pediatric counseling and testing (CT) as the entry point of a family screening strategy. The study was conducted at three community health facilities (General Hospital of Bonoua, Urban Health Facility of Koumassi and Urban Health Facility of Abobo Avocatier) in Abidjan, Côte d'Ivoire. The study on health care workers acceptability towards routine infant HIV testing was reported elsewhere [24]. Since 2004, those health centers offer comprehensive HIV/AIDS care and treatment program, including HIV voluntary counseling and testing services, PMTCT services, and antiretroviral treatment for children and adults infected by HIV.

Any child aged 6–26 weeks attending postnatal care at any pediatric service (immunization, weighing and consultation) in either three centers, was eligible for this study with four sequential contacts over a three-month period. Their parents/caregivers (mother, father, caregiver or legal guardian) were also eligible for this survey.

At the first contact, trained counselors offered systematic early infant diagnosis to index children and an HIV CT to their mother/parents/caregiver. Counselors translated and explained the study information sheet in mother -tongue to illiterate women. The process of the HIV testing method, return appointments, and provision of results was explained by trained counselors to each caregiver who agreed to participate in the study. An HIV serology was first performed for mothers who accepted their own test. Each mother was encouraged to discuss infant testing with the child's father or legal guardian before formal acceptance of EID. Mothers returned at home with the study information sheet and the consent form, in order to present them to their partner or the child's legal guardian.

At the second contact two weeks later, the mother's result was disclosed to her with confidentiality, if she was tested. We collected the parental consent for EID independently of the HIV mother's status. Then, we performed an HIV testing for all HIV exposed infants for those whose both parents (or legal guardian) had given written informed consent, as required by the Ivoirian Ethical Committee.

Children whose mother was either tested HIV-positive at the first contact or HIV-unknown first had a serology before HIV PCR testing. Children whose mother was tested as HIV-uninfected at the first contact were not tested at all, assuming that at this age (<6 months), the nosocomial risk of HIV infection by contaminated injections was low in this context. Real-time PCR was performed only in HIV-exposed infants (defined by a positive rapid test) [25], while adults underwent rapid HIV testing using two parallel assays (Determine®+Genie II®). The child's PCR result was disclosed with confidentiality to parents at the third contact (four weeks after the test), and children diagnosed HIV-infected were referred to the pediatric HIV care program. At the pediatric HIV centre (the fourth contact), free HIV care was offered. All family transport costs were supported by the project.

### Data collection

Standardized structured questionnaires were used at the first contact, to collect information on mother's acceptability of EID, then on parents'/caregivers' acceptability of HIV testing for both their infants and themselves. Parents/caregivers were asked about their socio-demographic characteristics, prior experiences with HIV testing and PMTCT services, how they felt about the option of infant testing, and reasons for accepting or declining infant testing.

Sub-samples of parents who refused and accepted infant HIV testing were interviewed at each pediatric health center by a public health sociologist, using a semi-directive grid tool to further understand the local interpretation of the acceptability of HIV testing in children, the mother's perceptions of EID, the reasons for accepting or refusing HIV testing for their infant, and the constraints and difficulties related to HIV early infant diagnosis.

### Data analysis

All children whose parents/caregivers received a proposal for a pediatric HIV CT were included in the analysis. First, descriptive baseline characteristics of children and caregivers were analyzed. Next, five outcomes were analyzed: 1) maternal acceptance of infant HIV-testing (defined as the formal mother's consent only; 2) adequate parent's acceptance of infant HIV testing (requiring both parents' signed informed consent: acceptance was deduced from the number of returned informed signed consent); 3) mother's and 4) father's formal acceptance of their own HIV testing; 5) parent/caregiver acceptance of the notification (defined as the proportion of caregivers who returned for infant test results, among those HIV tested).

These proportions were expressed as percentages and reported with their 95% confidence intervals.

Simple frequency distributions were calculated for responses collected from questionnaires. Associations between categorical variables were assessed using Pearson χ^2^ test. Univariate and multivariate logistic regression was performed to explore the correlates of first the “maternal acceptance”, then “both parents acceptance”, of infant HIV testing. Independent variables were clinical centre, mother's religion, ability to write, way of live, self-reported prenatal screening, self-reported prenatal status and the disclosure of their HIV status to their partners. A subset of 2962 children with completed mothers' informative records was used to perform this. For the adjusted analysis, we excluded variables with p-value>20% in univariate analysis. The final model was obtained using a backward-stepwise strategy with consideration for interaction and cofounding. The criterion for the statistical significance was set at an α of 0.05. Statistical analysis was performed using SAS 9.1 (TS1M3).

The qualitative analysis of parental interviews was done thematically by an inductive approach.

## Results

### Characteristics of mothers and infants

**Figure 1:**
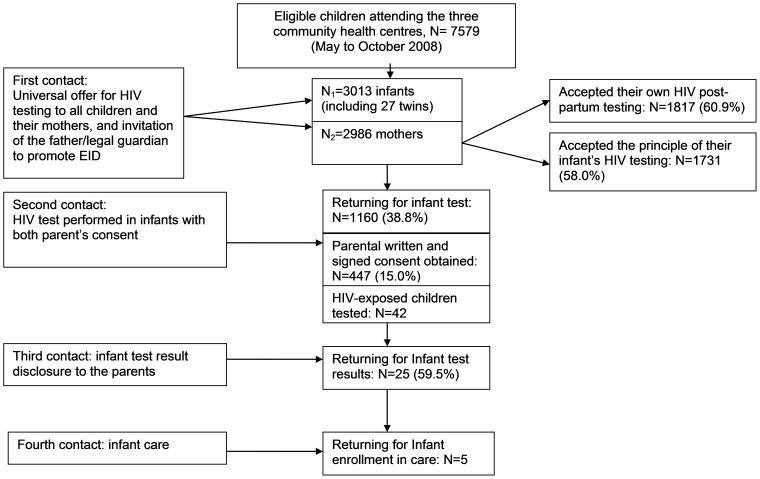
Flow diagram of the population selection after a universal offer for early infant HIV diagnosis (N = 3013).

From May to October 2008, among the 7,579 eligible children aged 6–26 weeks who attended pediatric services (immunization, weighing and consultation) at the three community health centers, 3,013 children (39.8%) born to 2,986 mothers who all agreed to participate in the survey, were offered HIV counseling and testing (Figure 1). At test offer, children had a median age of 3 months (inter-quartile range (IQR): 2–4 months), 27 were twins and 1520 (50.4%) were boys (Table 1). The reason of the first infant visit was immunization for 2434 children (80.8%), weighing for 529 (17.6%) and illness for 49 (1.6%).

**Table 1:** Children (n = 3013) and mothers (n = 2986) characteristics at inclusion.

Children characteristics	N (%)	Mothers characteristics	N (%)
	N = 3013		N = 2986
**Health facility**		**Health facility**	
Abobo-Avocatier	947 (31.4)	Abobo-Avocatier	940 (31.5)
Bonoua	747 (24.8)	Bonoua	734 (24.6)
Koumassi	1319 (43.8)	Koumassi	1312 (43.9)
**Sex**		**Can write**	
Male	1520 (50.4)	Yes	1653 (55.4)
Female	1485 (49.3)	No	1333 (44.6)
Missing	8 (0.3)		
**Breastfeeding since birth**		**Married**	
Yes	2978 (98.8)	Yes	1650 (55.3)
No	34 (1.1)	No	1336 (44.7)
Missing	1 (0.1)		
**Reason of the infant visit**		**Self-reported previous prenatal HIV screening**	
Immunization	2434 (80.8)	Yes	2370 (79.4)
Weighing	529 (17.6)	No	616 (20.6)
Pediatric consultation	49 (1.6)	**Self-reported knowledge of their prenatal HIV status**	
		Unknown	1096 (36.7)
**Mother-reported previous HIV infant diagnosis**		Known	1890 (63.3)
Yes	26 (0.9)	**Religion**	
No	2978 (98.8)	Christian	1852 (62.0)
Missing	9 (0.3)	Muslim	964 (32.3)
		Animist	156 (5.2)
		**Mode of life**	
		Alone	65 (2.2)
		With her partner	2018 (67.6)
		With her family/family in law	909 (30.2)

PEDITEST ANRS 12165 Study, Abidjan, Côte d'Ivoire, 2008.

Mothers had a median age of 26 years (IQR: 22–30 years); 55.3% were married; 67.6% lived with their partners; 55.4% could write; and 62.0% were Christian (Table 1).

### Maternal acceptance of infant HIV screening

**Figure 2:**
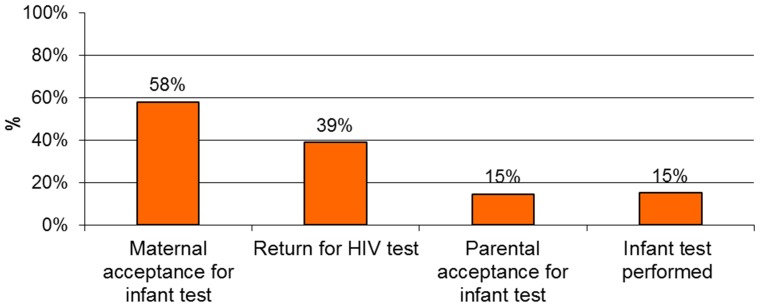
Rate of maternal acceptance and adequate parental acceptance for routine early infant HIV testing (N = 3013).

Among the 2,986 mothers included in the study, 1731 (58.0%: 95% CI: [56.2%–59.7%]) gave their own consent for their child to be HIV tested (Figure 1, Figure 2). A total of 2,506 mothers (83.9%) wanted to first get their partner's consent before accepting the infant's test and 51.5% thought that their partner would accept testing their child for HIV. Table 2 presents the correlates of maternal acceptance of EID, according to their self-reported prenatal HIV status: 55 mothers had self-reported that they were already previously identified as HIV infected during their pregnancy, of whom 35 (63.6%) accepted EID. Among the 20 (36.4%) mothers who refused the principle of EID despite the knowledge of their status, 18 (90%) self-reported that they had received antiretrovirals for PMTCT but were not on antiretroviral therapy.

**Table 2:** Correlates of maternal acceptance (n = 2962) of infant HIV testing (logistic regression).

Mother characteristics	Refusal (N = 1238)		Acceptance (N = 1724)		Univariate analysis			Adjusted analysis		
	N	%	N	%	OR	95%CI	p	aOR	95%CI	p
**Health facility**							<0.0001			<0.0001
Abobo Avocatier	540	57.8	393	42.1	1			1		
Bonoua	309	42.4	420	57.6	1.9	[1.5–2.3]		1.7	[1.4–2.1]	
Koumassi	389	29.9	911	70.1	3.2	[2.7–3.8]		3.2	[2.6–3.8]	
**Can write**	657	40.1	980	59.9	1.2	[1.0–1.3]	0.04	1.0	[0.8–1.2]	0.9
**Religion**							0.04			0.006
Christian	742	40.2	1104	59.8		1		1		
Muslim	433	45.0	529	55.0	0.8	[0.7–0.9]		0.7	[0.6–0.9]	
Animist	63	40.9	91	59.1	0.9	[0.7–1.4]		1.1	[0.8–1.5]	
**Mode of life**							<0.0001			0.15
Alone	22	33.8	43	66.2	1			1		
With her partner	898	44.8	1105	55.2	0.6	[0.4–1.0]		0.7	[0.4–1.2]	
With her family/family in law	318	35.6	576	64.4	0.9	[0.5–1.5]		0.8	[0.5–1.4]	
**Self-reported prenatal screening performed**	774	38.8	1222	61.2	1.5	[1.2–1.7]	<0.0001	NI	NI	NI
**Self-reported prenatal HIV status**							<0.0001			0.54
Unknown	514	47.2	576	52.8	1			1		
Not infected	704	38.7	1113	61.3	1.4	[1.2–1.6]		0.9	[0.6–1.2]	
Infected	20	36.4	35	63.6	1.6	[0.9–2.7]		1.2	[0.6–2.2]	
Disclosure of maternal prenatal HIV status to her regular partner	646	38.2	1043	61.8	1.4	[1.2–1.6]	<0.0001	1.2	[0.8–1.6]	0.22

PEDITEST ANRS 12165 Study, Abidjan, Côte d'Ivoire, 2008.

OR: Odds Ratio; aOR: Adjusted Odds Ratio. NI: not included.

In the univariate analysis, type of health facility, ability to write, being Christian, living alone, having been prenatally HIV tested, knowing their prenatal HIV status, and having disclosed their HIV prenatal status to their regular partner were independently associated with an increased maternal acceptance of infant HIV testing (Table 2). In the adjusted analysis, maternal acceptance of infant HIV testing was significantly lower in Muslim mothers and in mothers from the Abobo-Avocatier health facility, while the impact of other variables did not reach statistical significance.

### Formal parental acceptance for the infant HIV screening

Parental acceptance of infant HIV CT (Figure 1) was notably lower. Among the 2,986 mothers who were offered HIV EID, only 1160 (38.8%) returned at the second contact for infant testing, after discussion with their partner or legal guardian. Women who returned differed from those who did not returned on both religion and self-reporting of their prenatal HIV status. Indeed, 44.4% of Christian mothers returned versus 28.4 of Muslims and 37.2% of Animists (p<0.0001); In addition, 61.8% of self-reported HIV-infected mothers returned versus 37.4% of self-reported uninfected mothers and 40.2% of mothers unaware of their HIV status (p = 0.0006).

Only 447 infants had a formal and adequate parental/caregiver authorization to be HIV tested (of whom 435 mothers presented the signed, written informed of their partners, and 12 mothers were single or widowed).The rate of the formal and adequate parental acceptance was estimated to be 15.0% (95% CI: 13.7%–16.2%, Figure 1 and 2).

Correlates of formal parental acceptance of HIV infant testing are presented in Table 3.

**Table 3:** Correlates of adequate parental acceptance (n = 2962) of infant HIV testing (logistic regression).

Mother characteristics	Refusal (N = 2521)		Acceptance (N = 441)		Univariate analysis			Adjusted analysis		
	N	%	N	%	OR	95%CI	p	aOR	95%CI	p
**Health facility**							<0.0001			<0.0001
Abobo Avocatier	793	84.7	143	15.3	1			1		
Bonoua	544	75.2	179	24.8	1.8	[1.4–2.3]		1.5	[1.2–1.9]	
Koumassi	1184	90.9	119	9.1	0.5	[0.4–0.7]		0.6	[0.4–0.7]	
**Can write**	1372	83.8	266	14.2	1.3	[1.0–1.5]	0.02	1.1	[0.9–1.4]	0.4
**Religion**							<0.0001			<0.0001
Christian	1492	80.9	352	19.1	1			1		
Muslim	895	93.0	67	7.0	0.3	[0.2–0.4]		0.4	[0.3–0.5]	
Animist	134	85.9	22	14.1	0.7	[0.4–1.1]		0.6	[0.4–1.0]	
**Mode of life**							0.50	NI	NI	
Alone	52	80.0	13	20.0	1					
With her partner	1707	85.2	296	14.8	0.7	[0.4–1.3]				
With her family/family in law	762	85.2	132	14.8	0.7	[0.4–1.3]				
**Knowledge of prenatal HIV status**							0.56	NI	NI	
Unknown	924	84.6	168	15.4	1					
Known	1597	85.4	273	14.6	0.9	[0.8–1.2]				
**Self-reported prenatal HIV status**							<0.0001			<0.0001
Unknown	924	84.6	168	15.4	1			1		
Not infected	1567	86.3	248	13.7	0.9	[0.7–1.0]		0.9	[0.8–1.2]	
Infected	30	54.5	25	45.5	4.6	[2.6–7.9]		5.9	[3.3–10.6]	
**Disclosure of maternal prenatal HIV status to her regular partner**	1443	85.5	245	14.5	0.9	[0.8–1.1]	0.51	NI	NI	

PEDITEST ANRS 12165 Study, Abidjan, Côte d'Ivoire, 2008.

OR: Odds Ratio; aOR: Adjusted Odds Ratio. NI: Not included.

In the univariate analysis, the formal parental acceptance for HIV infant testing was not globally correlated with maternal knowledge of prenatal HIV status (14.6% versus 15.4%; OR: 0.9; 95% CI: [0.8–1.2]). However, mothers who self-reported as prenatally HIV-infected were significantly more likely to present formal parental consent to authorize infant HIV testing (45.5%) than mothers who self-reported as HIV-uninfected (13.7%) or as unaware of their HIV status (15.4%), (p = 0.0001). Final formal parental acceptance was not associated with the disclosure of maternal HIV status to partners. Infants of mothers attending the Koumassi health facility, infants of Muslim mothers, and infants of mothers living with partners or family had significantly lower formal parental acceptance rates than infants in other groups (Table 3).

In the adjusted analysis, the parental acceptance rate for HIV infant testing was strongly correlated with maternal self-reported prenatal HIV status: self-reported HIV-infected mothers were six times more likely to present the written and signed partner consent acceptance than mothers with self-reported unknown or negative prenatal HIV status (aOR: 5.9; 95% CI: 3.3–10.6, p = 0.0001). Being Muslim and attending the Koumassi health facility were also associated with a significantly lower acceptance rate, while attending the Bonoua health facility was associated with a significantly increased acceptance rate (Table 3).

### Family acceptance of routine HIV testing through pediatric point of care and HIV prevalence results

In our study, we offered maternal HIV counseling and testing to the 2,986 mothers of the included children, as a postpartum HIV test. Among them, 2,370 (79.4%) self-reported that they had been previously HIV tested during pregnancy; 1,890 (63.3% of the 2,986 mothers) knew their HIV status; 55 mothers had self-reported that they had been previously identified as HIV infected during pregnancy, and 36 (65.4%) of them had received antiretrovirals for PMTCT prophylaxis. Among the 55 infants reported by their mother to be HIV-exposed, 26 children (47.2%) had already been HIV tested at six weeks of age.

Overall, 1,817 mothers (60.9%; 95%CI: 59.1%–62.6%) accepted their own postpartum HIV test and 81 were identified as HIV-infected (4.5%; 95% CI: 3.5%–5.4%). Among the 81 HIV infected mothers, 27 (33%) had self-reported that they were HIV infected, 15 (19%) that they were uninfected and 39 (48%) reported an unknown HIV status.

A total of 46 fathers (1.5%) came to the health center for infant testing, of whom 35 (76.1%; 95%CI: 63.8%–88.4%) accepted their own HIV testing, and 2 (5.7%; 95% CI: 0–13.4%) were HIV-infected.

### HIV prevalence on children tested and access to HIV care

Among the 81 HIV-exposed infants, 42 underwent HIV testing (51.8%). Of these infants, 25 (59.5%) had mothers who returned for infant HIV test results. Among the 81 HIV exposed children, only 2 came from the pediatric ward and 79 from the immunization or weighing clinic; 26 (32%) were already HIV-diagnosed but none of them were already engaged in care at the time of the study. Five of the 42 tested infants were infected (11.9%; 95% CI: [2.1%–21.7%]) and all were then included in the pediatric HIV care program (Figure 1).

### Social determinants for accepting or refusing the early infant diagnosis

A subset of 35 parents with a postpartum HIV test result (of whom 15 uninfected mothers, 18 mothers with unknown HIV status and one discordant HIV-negative couple) were interviewed. Among them, 20 mothers (seven uninfected and 13 unknown status) and the discordant couple accepted the HIV test for their infants. Reasons given for accepting testing were as follows: “for their infant's health,” “to check her/his infant HIV status,” “to avoid HIV,” “to allow for early antiretroviral care if the infant test is positive,” and “to check the parent's HIV status.” All participating parents considered the father to be the chief of the family, and stated that the father's/couple's agreement about HIV infant testing would ensure the further stability of the couple.

Parents who refused infant HIV testing, (eight HIV-uninfected and five HIV-unknown mothers) mentioned that they refused infant testing for the following reasons: “the infant is too young to have a blood sample,” “he looks healthy,” “he is not ill,” “this is not useful as parents are healthy,” “they do not want to know if he is HIV-infected,” and “it is useless to know as there is no means to take care of HIV-infected children.” Different perceptions were reported: Mothers felt guilty and responsible for their infants' health, or felt the father should be the one to decide about HIV testing. They were afraid of the social stigma that might result if the child testing HIV-positive, and cited social risk for the mother, risk of mother and infant rejection by the father and the family, and additional costs for the mother to take care of her infant alone. They also raised the fear of being unable to administer antiretroviral therapy, using statements including “afraid of not knowing how to give the antiretroviral treatment to her child” and “afraid of opportunistic infections and death.”

The main and common motivations of rejection of EID were the “apparent good health of child” and the “special status accorded to the HIV infection”. But, although common, these reasons do not follow the same logic. Mothers who accepted the test because the “child was in good health” would like to be conservative and believe that the result will be negative. When mothers refused the test because the “child was in good health”, they thought that if parents look healthy, it is unacceptable to offer EID for children. For example, “[…] Why perform an HIV test … we (parents), are healthy and are asked to consent to HIV testing for children. I cannot accept that”. Mothers also argued that their child being overweight was not only an indicator of good health but also a guide to perform or not an HIV test. When the child has a “shiny” appearance, mothers were not interested in the HIV test because they had no doubt about its probable HIV infection.

Parents who refused EID did not accept the “special status accorded to the HIV infection” by health workers. They believed that this status had a negative impact on their perception of the disease. The “special status accorded to HIV infection” (unlike other diseases) increases the fear of parents and led mothers to refuse any intervention related to HIV/AIDS. Moreover, as those who accepted the pediatric HIV test, mothers who refused would like to be guided in their decisions by health workers. These excerpts from interviews further illustrate the thinking of respondents: “When my children are sick, the doctor is doing for them what he thinks is good without question … even when he asks, it is whether we can afford the medicine… but for HIV/AIDS, he still asks questions to scare us … we know nothing about this, that's why we came to the hospital …” Miss F. 29 years, (refused EID, unknown HIV status, customary marriage).

Some parents who accepted HIV EID, as those who refused, wondered about the need and necessity to have their consent before infant testing: “Why for other diseases, they do not asked if I want to or not?… and then I hear about HIV/AIDS, it is long, they ask if I want to or not, why is it not the same and it's only about HIV/AIDS that we have questions. I think all of this is scary and when it is like that […] people refuse and they are right.” Miss C. 32 years (accepted EID, uninfected HIV status, customary marriage).

For these parents, if the formal consent is not usually required before treating other diseases, then no written consent should be required prior to any intervention in the case of HIV/AIDS. According to them, the procedure that subordinates the HIV infant diagnosis to the prior approval of his both parents, could explain the reluctance of some parents to accept it. In the opinion of respondents, it is the role of health professionals to make every effort to improve the health of the patient, and not ask its opinion to carry out the care required by the diagnosis. However, it is important to inform fathers on their infant if the HIV test result is positive.

## Discussion

Limited access to early infant HIV diagnosis in resource-limited settings is a major barrier to the early antiretroviral treatment initiation that has been recommended since 2008 [8], [9], [10], [13], [26]. Many HIV-infected children are either never identified or are lost before they can be enrolled into care. The scaling up of HIV testing programs has many social and community implications in Africa. However, few studies have analyzed the acceptability of this public health strategy and social barriers to early infant diagnosis implementation. Despite the growing pediatric epidemic, there are still few programs for the implementation of routine HIV infant testing, and those that have been reported were conducted in high-prevalence settings [16], [17], [18], [27], [28], [29]. Our study took place in Abidjan, Côte d'Ivoire, a setting with the highest estimated HIV seroprevalence in West Africa (7.4%), but with a low-intermediate HIV prevalence at the country level (3.4%) compared to other sites in Africa [19], [21] and with poor coverage of PMTCT services [23]. As elsewhere in Africa, access to pediatric antiretroviral care occurs too late in Abidjan compared to the WHO recommendations, with a median age of 63 months at antiretroviral treatment initiation [12]. In this context, we highlight the social difficulties in accessing early infant diagnosis in primary health care services.

First, we report that, although the principle of routine infant HIV testing was accepted by 58% of the mothers before discussion with their partners, only 39% of mothers returned for the second contact for infant testing. This low return rate and moderate rate of mother's acceptance could be explained by the mother's feeling of not being at risk of acute infection because of a recent HIV negative test during gestation, or the denial of illness. In addition, the care of healthy children is not yet a habit in Africa. Blood samples in healthy infants are often misunderstood and not accepted by mothers.

Second, adequate parental acceptance of routine infant HIV testing is low, estimated to be only 15% at the community level. This low final parental acceptance rate seems to be mainly due to the lack of formal paternal consent. The need for partner consent for infant testing was expressed by more than 80% of mothers; after maternal discussion with partners, only 15% of infants had an HIV test formally authorized by both parents. This highlights the key role of infant fathers in decision-making related to family health, an apparently incontrovertible social value in Côte d'Ivoire, in keeping with the statements of the Ivoirian National Ethics Committee, and in reference to the law of the civil code n°70–453 of 3 August 1970 on minorities in Cote d'Ivoire. Paternal agreement was also a key element in mother/infant return for receipt of test results and in subsequent access of antiretroviral treatment for HIV-infected children, as this was similarly reported for infant feeding choice in this context [30].

The issue of parental informed consent is complex, but crucial to improve uptake of infant HIV testing and care. In our study, although written informed consent from fathers or legal tutors was required in a population in which 45% of women were unable to write, the parental acceptance of EID was not differ according to this, adjusted on others variables (p = 0.4). The active father's refusal was not explored, and we did not assess the ability of mothers to explain EID or the consent form to their partners. Additionally, written informed consents were not signed at the hospital, in front of the physician to insure that mothers and fathers had a good understanding of EID before acceptance. Only 1.5% of fathers came to the health center for infant testing, but these fathers demonstrated high uptake of their own HIV testing. It is difficult to distinguish between the roles of partner refusal, maternal self-stigmatization, and other barriers to parental acceptance, and this needs further investigations. In fact, when the joint parental consent is formally obtained, we guess that is also the marker of the good communication between parents about disclosing their own HIV status: indeed, mothers who reported to have disclosed their own HIV status to their partner accepted significantly more often testing their infant than those who had not disclosed it, although this was not significant in the adjusted analysis, possibly due to a lack of statistical power. The question about the involvement of father's in HIV family care in Africa remains open and cannot be closed without specific communication strategy for men. Our study is indicative that consent from both parents in a context with low father involvement in child health care may not be feasible and maternal consent may be sufficient to test children. However, the father's consent or at least information could become important if the child is infected and therefore needs to be treated with antiretroviral therapy as it is recommended since 2010. We feel that previous father consent would also facilitate the access to pediatric HIV care, although, we were not able to provide data on this point.

We felt that in this prevalent context, an opt-out option to routinely perform the postnatal early HIV infant testing without any formal parent consent as it was suggested elsewhere in Africa [31], would not seem to be an option in this context because if the infant is diagnosed HIV-infected, this would require ideally both parents adherence and thus awareness of their infant's HIV status in order to accept to treat her/him. Indeed, the lack of parental, and particularly paternal, awareness about pediatric HIV services could be an important barrier to access to HIV treatment for children. The main difficulty would be to convince fathers to come to clinical centres. It is not easy to obtain simultaneous agreement of both parents. The main question is how to approach fathers without stigmatization or reject for mothers, and without dispute between the couple? That requires a good knowledge and practice of familial HIV counselling and testing by health workers and counselors.

Best practices to communicate with men about HIV testing and to involve them in HIV family care in Africa remain crucial [30], [32]. In Uganda, to improve identification and treatment of HIV infected children, Baylor implemented a program in adult ART clinics entitled “Know Your Child's HIV Status”. Within this campaign, a total of 4,737 people were HIV tested, of whom 92.1% were children [33]. That raises the possibility to improve infant testing strategies by enrolment of a network of adults, especially fathers.

Third, adequate parental acceptance of EID was significantly higher among mothers who self-reported as HIV-infected prenatally, compared to mothers who reported themselves as HIV-uninfected or unaware of their HIV status prenatally. HIV-infected mothers informed prenatally of their HIV-status were probably more aware about interventions for PMTCT and better prepared to accept postnatal infant testing. In addition, reasons for refusing HIV testing were highly correlated with lack of knowledge of PMTCT interventions among HIV-negative or HIV-unknowing mothers [27], [34], [35], [36]. In urban areas of Cameroon, HIV-infected women diagnosed sufficiently early during pregnancy opt to benefit from EID whatever their socio-economic, marital or disclosure status [36]. These findings prove the importance and the role of HIV CT in maternity clinics to primarily improve both the coverage of prenatal HIV testing and the community knowledge about pediatric HIV prevention and care. However, the real social impact of links between PMTCT programs and pediatric HIV programs should be explored through studies on the knowledge, attitudes and practices of mothers, fathers, community leaders and health staffs on this topic [23].

EID programs in many countries may improve the identification of HIV-infected infants, and should be considered in the context of the underlying HIV prevalence. In all countries, children who are hospitalized represent an opportunity for routine HIV testing. In the high-prevalence setting of Lusaka, Zambia, the routine offer of HIV testing to hospitalized pediatric inpatients identified large numbers of HIV infected children [16]: among 17,003 hospitalized pediatric inpatients, HIV testing was accepted by 87% of caretakers of children not previously tested. The highest testing rates were found among children <12 months of age and symptomatic. Similarly, at the Mulago Hospital in Uganda, caretakers agreed to HIV testing for 92.8% of 9687 hospitalized children. Overall HIV prevalence among these children was 12.4%, and was highest on the nutrition ward (30.8%) and in children who had a CD4 percentage <20% [27]. HIV-testing for hospitalized children and their caretakers could identify a significant number of HIV infected children but more than half of the children had advanced HIV disease and therefore late access to antiretroviral care. In our study, children from the primary health services were mainly asymptomatic (98%), so we could not access the acceptability rate among those with symptomatic HIV disease.

Acceptance of EID with unknown HIV-exposure status in our study was 52% for mother and 15% for both parents. This is much lower than that observed in other high-prevalence settings. In KwaZulu Natal, South Africa, where the HIV prevalence rate is approximately 39% among pregnant women and the PMTCT coverage up to 95%, the routine EID of all infants at immunization clinics was found to be acceptable and feasible as a means for early identification of HIV-infected infants [29]. Of 646 mothers bringing infants for immunization, 90.4% accepted infant HIV testing, and 56.8% of these subsequently returned for results. This difference with our findings could be explained by the gap between the two countries in HIV prevalence, PMTCT coverage and legal requirements on children testing.

Proactive EID in children known to be HIV-exposed would probably be most efficient in countries where the PMTCT coverage is high. However, the uptake of HIV postnatal testing remains low even in these settings. Parental attitudes need to be taken into account to ensure the success of HIV testing for children and their caregivers. In the United Kingdom, 73% of known HIV-infected mothers did not accept an offer of HIV testing for their infants. Primary reasons for testing refusal included the perception that a child who looks to be in good health cannot be infected with HIV, the fear of disclosure to others, and the fear of feeling guilty if the child was found to be positive [35]. Similarly, in Blantyre, Malawi, uptake of testing was lower than expected, with only 49% of known HIV-infected mothers presenting for infant testing at six weeks postpartum. Barrier to testing in this setting included poverty (lack of money for transport to primary health care clinic), stigma, especially when mothers had not disclosed their status, problems with identifying exposure status due to disconnections between mother and infant health records, and lack of awareness of infant testing and treatment availability in the community [37].

In 2008, the links between PMTCT programs, infant testing programs and pediatric care HIV programs were not effective because of poor referral systems [23], [38]. The necessity to improve them is evident. In our study, the rate of effective 6-weeks infant testing remained low even among self-reported HIV exposed children. Indeed, among the HIV-exposed infants self-reported by their mothers, the coverage rate of early infant diagnosis was 47% at baseline. After the implementation of the routine testing proposal, the coverage of EID rose only to 52% in HIV exposed-infants. This 5% increase in the coverage rate is useful, but remains insufficient to ensure widespread access to early pediatric HIV care in our low-intermediate prevalence setting. In addition, despite improvement in infant testing coverage, many women and children were lost to follow-up between HIV testing and receipt of test results [13]. These results are similar to those reported in high-prevalence settings. In Tanzania, the implementation of an early infant diagnosis pilot program showed that among the 510 HIV-exposed infants identified from health facilities, 87% of caregivers accepted infant testing, of whom 55% returned to receive PCR test results [17]. This is also similar to the 57% reported in South Africa [29].

In addition, data are needed to inform how HIV referral systems could improve identification of HIV-exposed and HIV-infected infants. Indeed, the referrals between PMTCT programs, HIV testing programs and pediatric care centers are often limited by lack of notification and poor health information systems. In Malawi, challenges identified with this system included an incompatible patient identification pattern, cumbersome documentation methods, and lack of systematic collaboration among programs, resulting in difficulties identifying and tracing patients. Referral procedures are variable among each health system, and staff is not usually trained on this topic in Abidjan [39]. The need of insuring infant tractability by an electronic identification schema should be promoted to guarantee best clinical practices for early HIV infant diagnosis. In Mozambique, improving the health system information by an electronically network (Expedited Results System) between remote districts and two central laboratories has reduced the delay in returning EID results and thus treatment initiation of HIV infected children [40]. This system contributed to a 60% increase in HIV infected children enrolling in ART [30], [31], [32], [33],[41].

Finally, among the 3,013 infant offered HIV testing in our study, 81 were found to be HIV exposed (2.7%) and five (12% of those tested) were identified as HIV-infected and subsequently treated. This very high prevalence in children compared to adults (3.4%) shows the necessity to better organize the early identification of HIV exposed children in Cote d'Ivoire. It will be possible trough improvement of links between six complementary and parallel programs: PMTCT, HIV pediatric care, HIV adult care, laboratories, health system information and community outreach programs.

However, the question of cost-effectiveness of infant routine HIV testing in low and medium HIV prevalence countries is important to explore. Screening infants with rapid HIV testing before DNA-PCR, as we did in our study, was shown to be cost-effective in infants aged three months or older in Uganda. Incorporating rapid HIV tests into early infant testing programs could improve cost-effectiveness and reduce program costs [39], [42], [43].

Early infant diagnosis of HIV infection at the primary care level in a resource-poor setting is challenging. In an intermediate HIV prevalence country, it could be helpful to identify HIV-exposed infants through maternal HIV testing when their mother has not been HIV-tested prenatally. In addition, it is also crucial to ascertain the maternal HIV status, as mother health is a key determinant of child health. Finally, also involving fathers in a comprehensive family approach would be ideal to ensure the child care.

HIV prevention programs on both a community and healthcare worker level could be carried out to promote prenatal HIV testing, including also couples and partner involvement in this process. To capture those who have refused the prenatal testing intervention, other delivery and postnatal opportunities should be also promoted at each point of care. In addition, we need to improve the acceptance of each step of the overall testing strategy. Indeed, despite an overall good coverage of the prenatal HIV testing strategy, the post-test acceptance rate of this prenatal testing still could be improved. Finally, it is crucial to insure the correct and effective delivery of PMTCT interventions and antiretroviral when needed to build a sustainable trust at a community level.

To improve EID parental consent, it is important to develop a community outreach program on children and HIV, including HIV prevention, voluntary counseling and testing for fathers and mothers with children less than five years, and target EID for any exposed children. But it is important to insure that antiretroviral treatment (including PMTCT) for adults and children is available in the community or district level. Also, in the context of HIV pediatric elimination campaign, one might consider merging this HIV community outreach program with the immunization program campaign for children under five years, as this was successfully reported in the Southern African context [28]. The advantage of such a public health program would be the pooling of expertise of the health system, the possibility of reaching almost all the target population of children under 5 years and the reduction of direct costs. But the feasibility and acceptability of this intervention should be discussed and tested in a pilot intervention in intermediate HIV prevalence country.

In conclusion, the implementation of routine HIV testing of asymptomatic infants attending primary health care services in Abidjan was poorly accepted at the community level in 2008. The lack of involvement of fathers, who represent the social and financial authority, appears as the main weakness in this public health strategy.

Successful identification of HIV-infected children requires each mother-infant pair go through a critical pathway, including prenatal maternal HIV testing, post-test counseling, adherence to PMTCT interventions, and postnatal infant diagnosis with receipt of infant test results. In this evaluation of a routine infant HIV testing program in Abidjan, Côte d'Ivoire, key challenges included the large proportion of parents, especially fathers, not accepting infant testing, and the large proportion of caregivers not returning for test results. Patterns of parental consent need to be understood in specific settings, in the context of local HIV prevalence and HIV care practices. In the low-prevalence context of Côte d'Ivoire, routine infant HIV testing in immunization clinics was not efficient in targeting HIV infected children because of the small number of HIV-infected children. Therefore, an active linkage between PMTCT services and postnatal points of care should be improved to target more specifically the truly HIV-exposed children, to promote early diagnosis of all infected infants. Community mobilization and awareness by health staff about pediatric HIV screening is urgently needed to maximize PMTCT coverage, postnatal diagnosis and roll-out access to pediatric antiretroviral therapy in West Africa.
